# Investigation of different commercial fining agents by SDS-PAGE and immunoblot

**DOI:** 10.1186/2045-7022-1-S1-P9

**Published:** 2011-08-12

**Authors:** Marina Deckwart, Carsten Carstens, Markus Fischer, Angelika Paschke-Kratzin

**Affiliations:** 1 University of Hamburg, Institute of Food Chemistry, Hamburg, Germany

## 

Allergenic fining agents and processing aids from hen's egg and cow's milk used in wine production are hidden allergens and could demonstrate a health threat for allergic persons. Hence, the European parliament adopted Directive 2003/89/EC amending 2000/13/EC to declare ingredients, contaminations and processing aids which are known to trigger allergic reactions. The Amendment Directive 415/2009/EC excludes the labeling of wine which are processed with eggs or milk and products thereof until the 31st of December 2010.

In 2006 Rolland et al. did a double-blind, placebo-controlled trial and basophil activation analysis about potential food allergens in wine including 5 egg-allergic and 1 milk-allergic patients. Among others they collected 24 egg white-fined or whole egg-added red wines, 34 milk-fined white wines and 25 casein-fined white wines. In this study no anaphylaxis was induced by wine consumption. But adverse reactions against treated wines could not be excluded. Due to the rarity of adult milk or egg-allergic patients reliable statistical analysis of allergic reactions against milk or egg-fined wines is inhibited.

Casein, ovalbumin/hen's egg white and lysozyme products of different commercial producers were investigated by SDS-PAGE and immunoblot. These investigations show that every product contains beside casein, ovalbumin or lysozyme other milk or egg allergens respectively in different amounts. The investigations show the necessity to have analytical methods for determination of residues of fining agents or stabilizers in wine which are able to detect not only casein, ovalbumin and lysozyme but also take into account the other milk and egg allergens. This could be guaranteed by certain antibodies used in ELISA systems binding not only to casein, ovalbumin or lysozyme but also to the other allergens in processed casein, ovalbumin or lysozyme for wine production. Therefore polyclonal antibodies raised against fining agents casein and ovalbumin and stabilizer lysozyme of one fining agent producer were used in this study.

